# Supplementary material for: Hybrid TiO2/Al2O3 nanolayer overcoating enhances dental lithium disilicate glass-ceramics acid resistance and surface mechanical properties
Source: Nat Commun. 2025 Nov 21;16:11530. doi: 10.1038/s41467-025-66560-8 (PMC12748710; doi:10.1038/s41467-025-66560-8)
Supplement: Supplementary file 1 — Supplementary Information [file 41467_2025_66560_MOESM1_ESM.pdf]

Supplementary Information

Hybrid TiO_2/Al_2O_3 nanolayer overcoating enhances dental lithium disilicate glass-ceramics acid resistance and surface mechanical properties

Xiaoxuan Zhang[1, †], Bin Zhang[2, †, *], Fanchun Meng[2], Xiangyu Zhao[1], Ruilin Liu[2], Meixian Liu[1], Huifei Li[1], Yuan Zhang[1], Feng Wu[1], Jin Bai[2], Zhuoqun Yan[3], Xiuyun Ren[1], Yong Qin[4, *], Xing Wang[1, 5, *]

[1]Shanxi Medical University School and Hospital of Stomatology, Taiyuan 030001, China.

[2]State Key Laboratory of Coal Conversion, Institute of Coal Chemistry, Chinese Academy of Sciences, Taiyuan 030001, China.

[3]Innovation Center, Upcera Co., Ltd., Shenyang 110000, China

[4]Prof. Y. Qin. College of Materials Science and Engineering, Qingdao University of Science and Technology, Qingdao 266042, China.

[5]Shanxi Province Key Laboratory of Oral Diseases Prevention and New Materials, Taiyuan 030001, China.

†These authors contributed equally: Xiaoxuan Zhang and Bin Zhang.

E-mail: wangxing@sxmu.edu.cn; zhangbin2009@sxicc.ac.cn; qinyong@qust.edu.cn.

Supplementary Methods

Corrosion tests

Corrosion tests were conducted according to ISO 6872:2024. Samples with at least 30 cm^2 of exposed surface area were prepared for the tests. The samples were cleaned in deionized water using an ultrasonic bath, dried in an oven at 150 °C for 4 h, and weighed to the nearest 0.1 mg. They were then immersed in 100 mL of 4% acetic acid solution and placed in an oven at 80 °C for 16 h. Afterward, the samples were rinsed with double-distilled water (ddH_2O), dried in an oven at 150 °C for 4 h, and reweighed. The mass loss ($\mu g\ cm^{-2}$) calculation formulas are as follows (3):

$$\text{Mass loss} = \frac{WB\text{-}WA}{SA} \quad (3)$$

WB is the mass of the specimens before the chemical solubility challenge (ug); WA is the mass of the specimens after 16 h of challenge and after drying (ug); SA is the total surface area of specimens (cm^2).

Dynamic corrosion tests were performed at a standard temperature of 37 °C. Samples were immersed in 4% acetic acid solution, which was renewed every 24 hours. The release of Li was measured by ICP-MS, and the surface morphology of the samples was characterized by FEGSEM at 24, 48, 72, and 96 hours after immersion.

In-vitro cytotoxicity studies

L-929 mouse fibroblasts were cultured in DMEM containing 10% FBS and 1% penicillin-streptomycin mixture. When the cells reached 90% fusion, they were digested with trypsin, resuspended with culture medium, and added into 96 well plates according to 1×10^4 cells per well to incubate for 24 h. The LDGC, Al_2O_3@LDGC,

TiO_2@LDGC, TiO_2/Al_2O_3@LDGC were ultrasonically washed in anhydrous ethanol for 20 min, rinsed with PBS, and finally incubated in DMEM at 37 °C for 24 h to obtain the extraction solution. The medium was discarded from the 96-well plate, and 200 μL of extraction solution was added to each well. The cells were randomly divided into five groups, with 3 replicates per group. The groups were as follows: blank group (containing only DMEM), LDGC extract group, TiO_2@LDGC extract group, Al_2O_3@LDGC extract group, TiO_2/Al_2O_3@LDGC extract group. After the cells were cultured at 37 °C for 48 h, the extraction solution was replaced with medium containing the CCK-8 reagent. After 30 minutes, the absorbance at 450 nm was measured using a microplate reader (SpectraMax i3, Molecular Devices, USA). For qualitative cytotoxicity analysis, a LIVE/DEAD™ Viability/Cytotoxicity Stain Kit was used to stain the samples. Live/dead fluorescent images were captured using a fluorescence microscope (IX51, Olympus, Japan), where live cells were stained green and dead cells were stained red.

2. Supplementary Figures

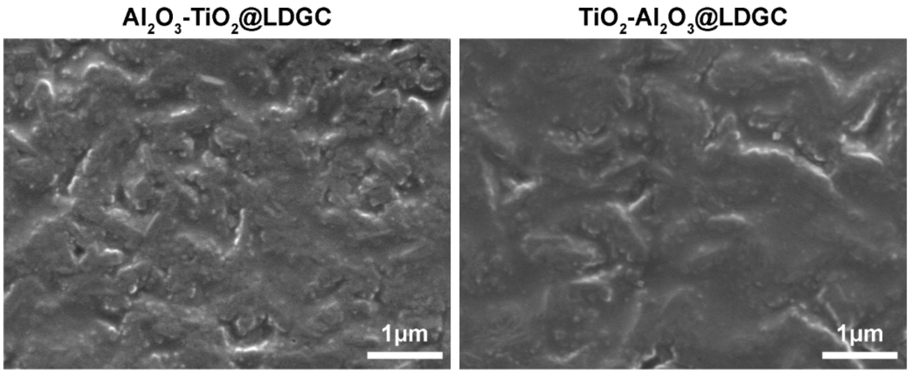

Supplementary Figure 1 | SEM images of the Al_2O_3-TiO_2@LDGC and TiO_2-Al_2O_3@LDGC.

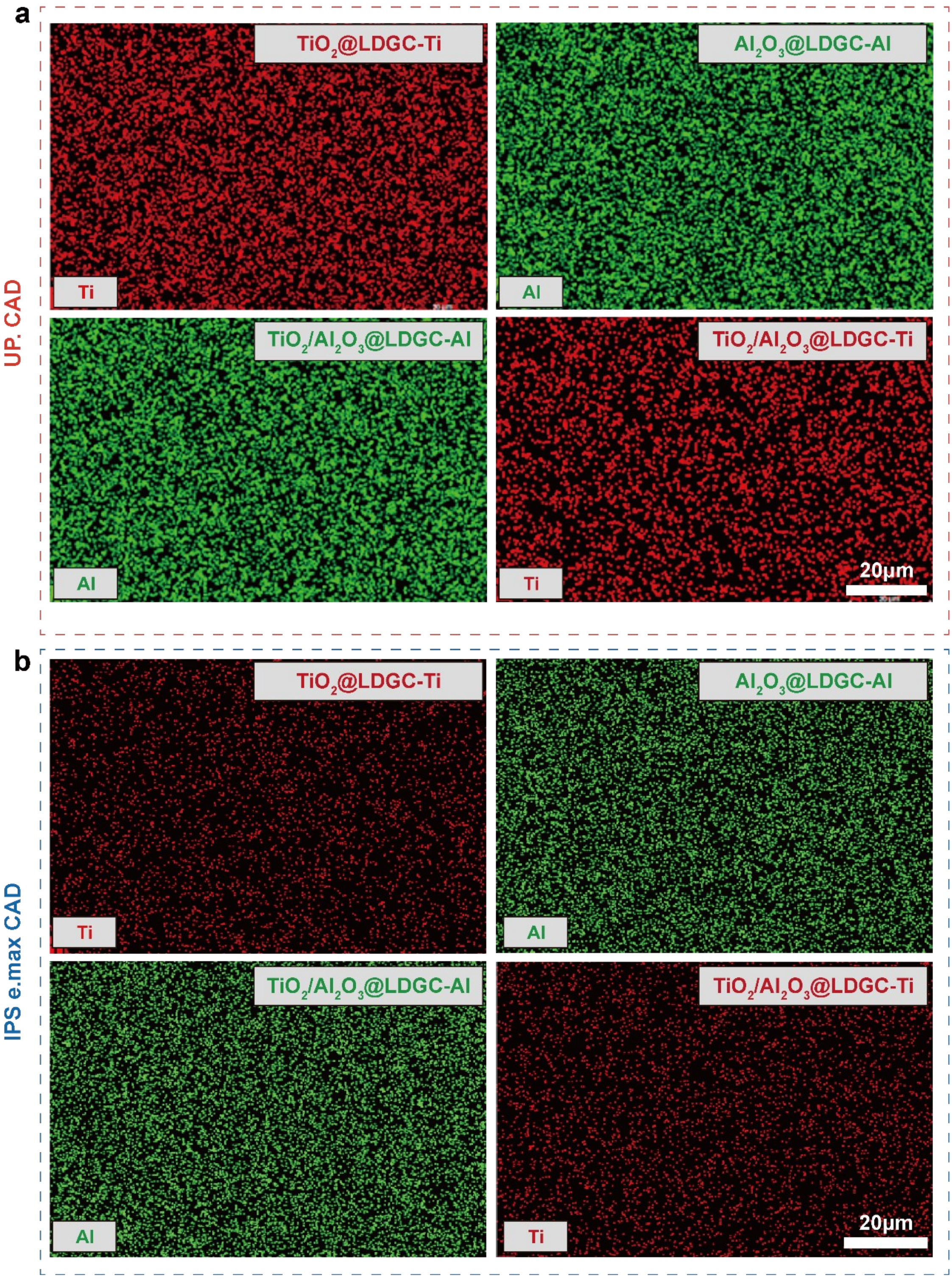

Supplementary Figure 2 | EDS analysis. (a) UP. CAD. (b) IPS e.max CAD (Green: Al; Red: Ti).

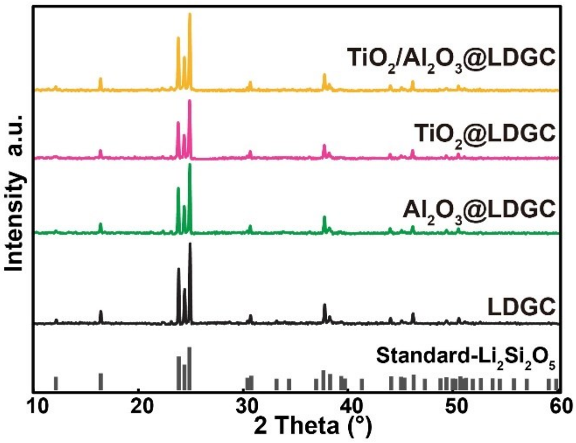

Supplementary Figure 3 | The X-ray diffraction results of LDGC, TiO_2@LDGC, Al_2O_3@LDGC, TiO_2/Al_2O_3@LDGC. Source data are provided as a Source Data file.

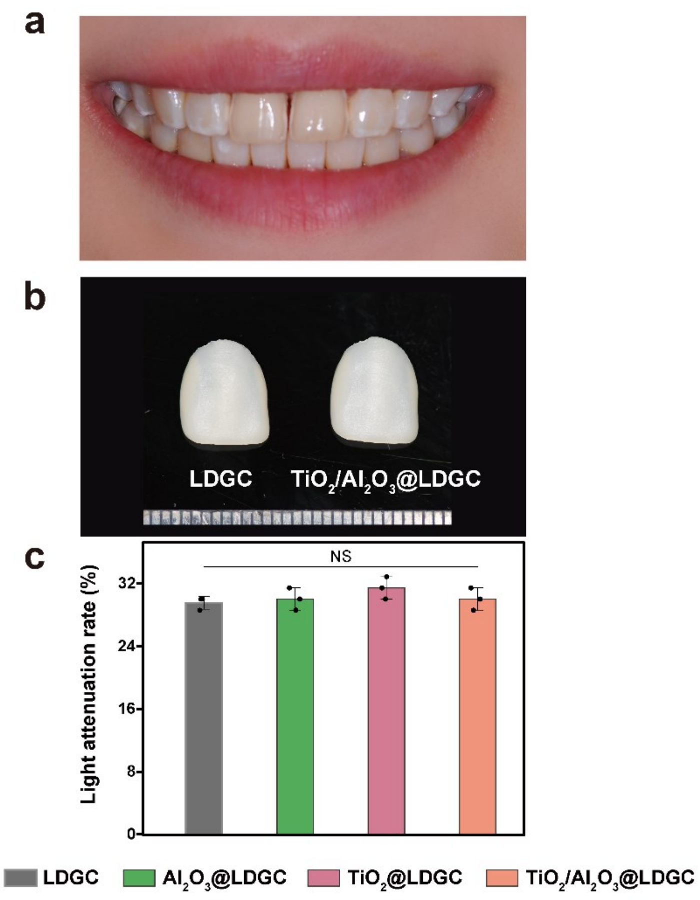

Supplementary Figure 4 | Aesthetic data. (a) Photos of teeth without veneers; (b) Photos of veneers; (c) Light attenuation rate of LDGC, TiO_2@LDGC, Al_2O_3@LDGC, TiO_2/Al_2O_3@LDGC. Statistical significance was analyzed using one-way ANOVA with Tukey's multiple comparisons test. NS is not significant. Data are presented as mean ± s.d. of n = 3 biological replicates. Error bars represent s.d.. Source data are provided as a Source Data file.

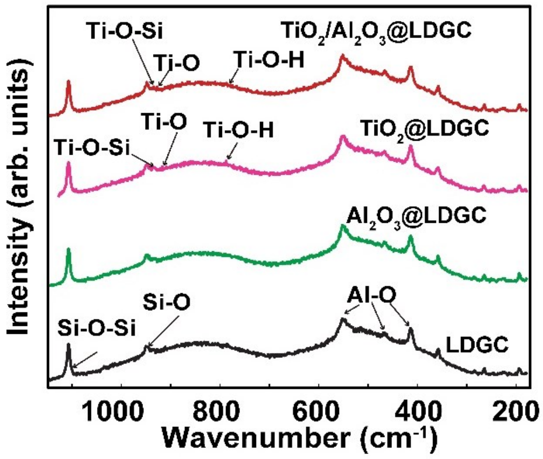

Supplementary Figure 5 | Raman spectroscopy detection of LDGC, TiO_2@LDGC, Al_2O_3@LDGC and TiO_2/Al_2O_3@LDGC. Arrows point to the corresponding peaks. Source data are provided as a Source Data file.

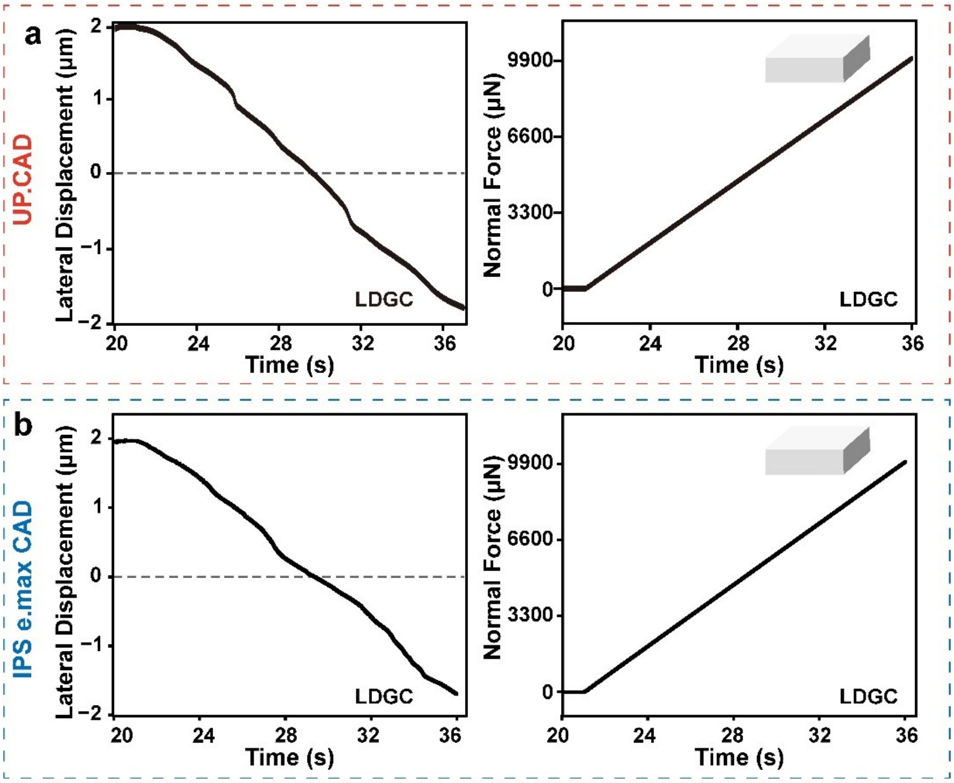

Supplementary Figure 6 | Nano-scratch test for LDGC of the time-lateral displacement data and the time-normal force: (a) UP. CAD; (b) IPS e.max CAD. The insets in panels a-b are schematic diagrams of LDGC. Source data are provided as a Source Data file.

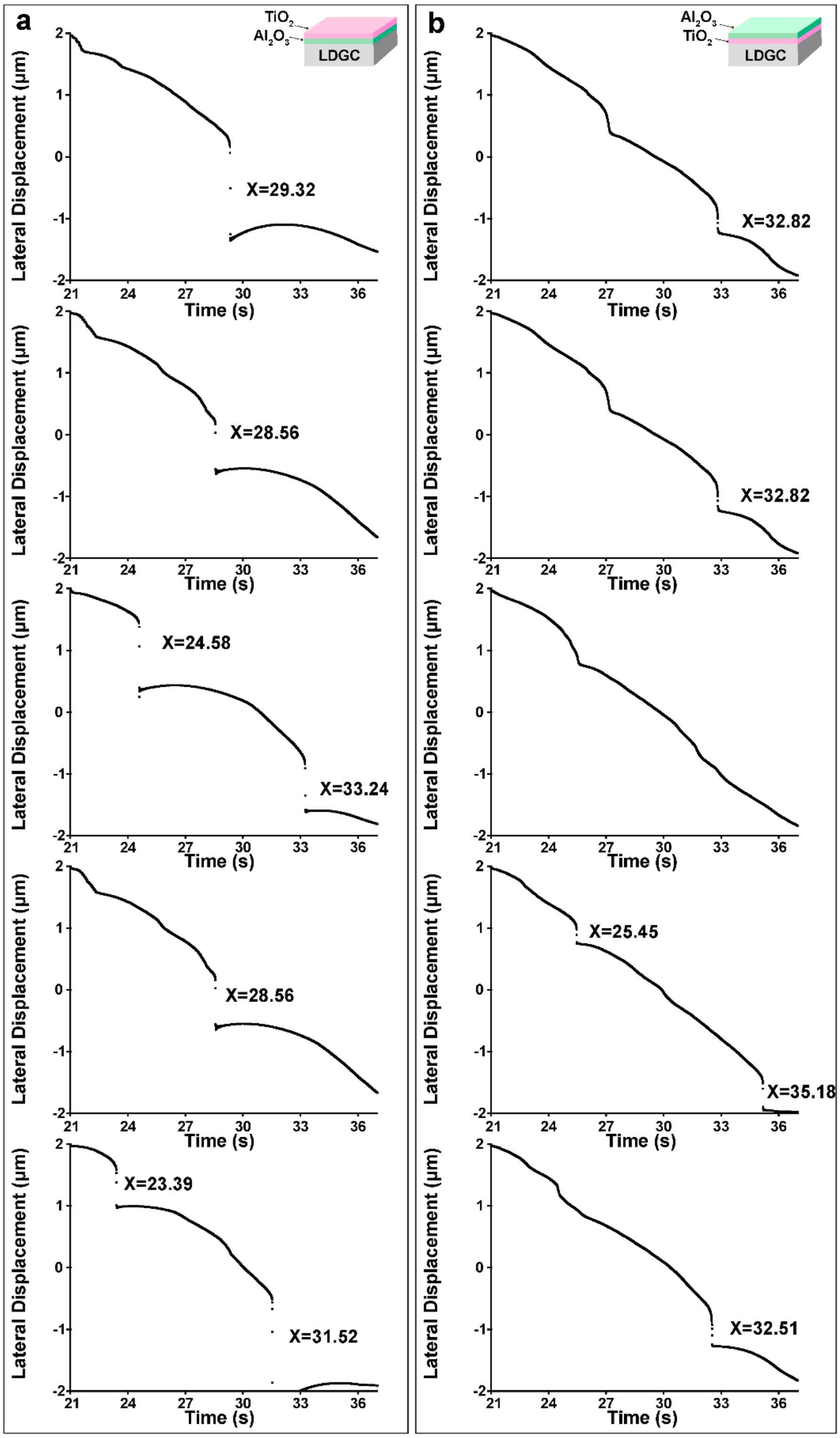

Supplementary Figure 7 | Nano-scratch test for double nanolayer (UP. CAD). (a) Al_2O_3-TiO_2@LDGC; (b) TiO_2-Al_2O_3@LDGC. The insets in panels a-b are schematic diagrams of LDGC coated with different thin films. Source data are provided as a Source Data file.

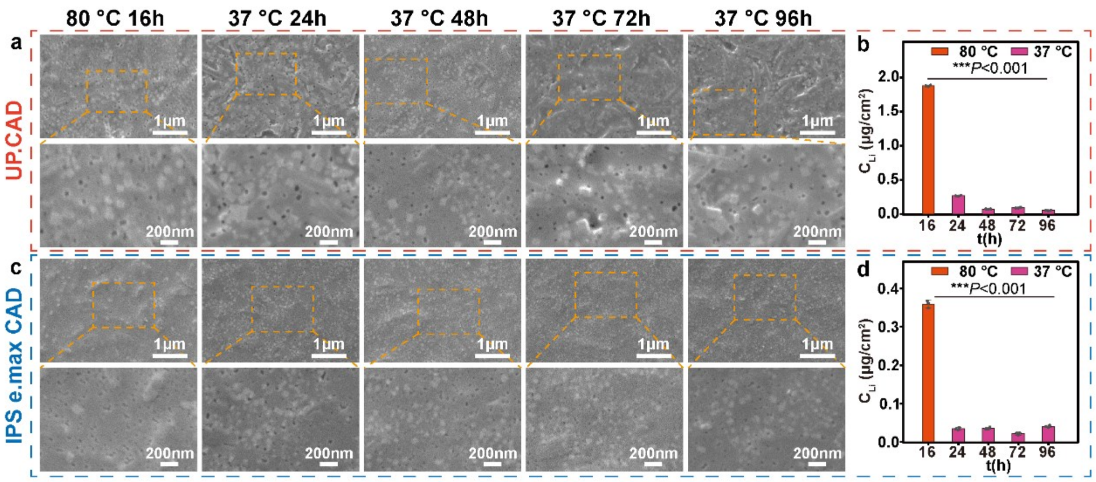

Supplementary Figure 8 | Corrosion tests were conducted by immersing samples in a 4% acetic acid solution. SEM of LDGC soaked in 4% acetic acid (a) UP.CAD, (c) IPS e.max CAD; Li-ion release from LDGC by ICP-MS (b) UP.CAD, (d) IPS e.max CAD. Statistical significance was analyzed using one-way ANOVA with Tukey's multiple comparisons test. P(80 °C-37 °C) < 0.001. Data are presented as mean ± s.d. of n = 3 biological replicates. Error bars represent s.d.. Source data are provided as a Source Data file.

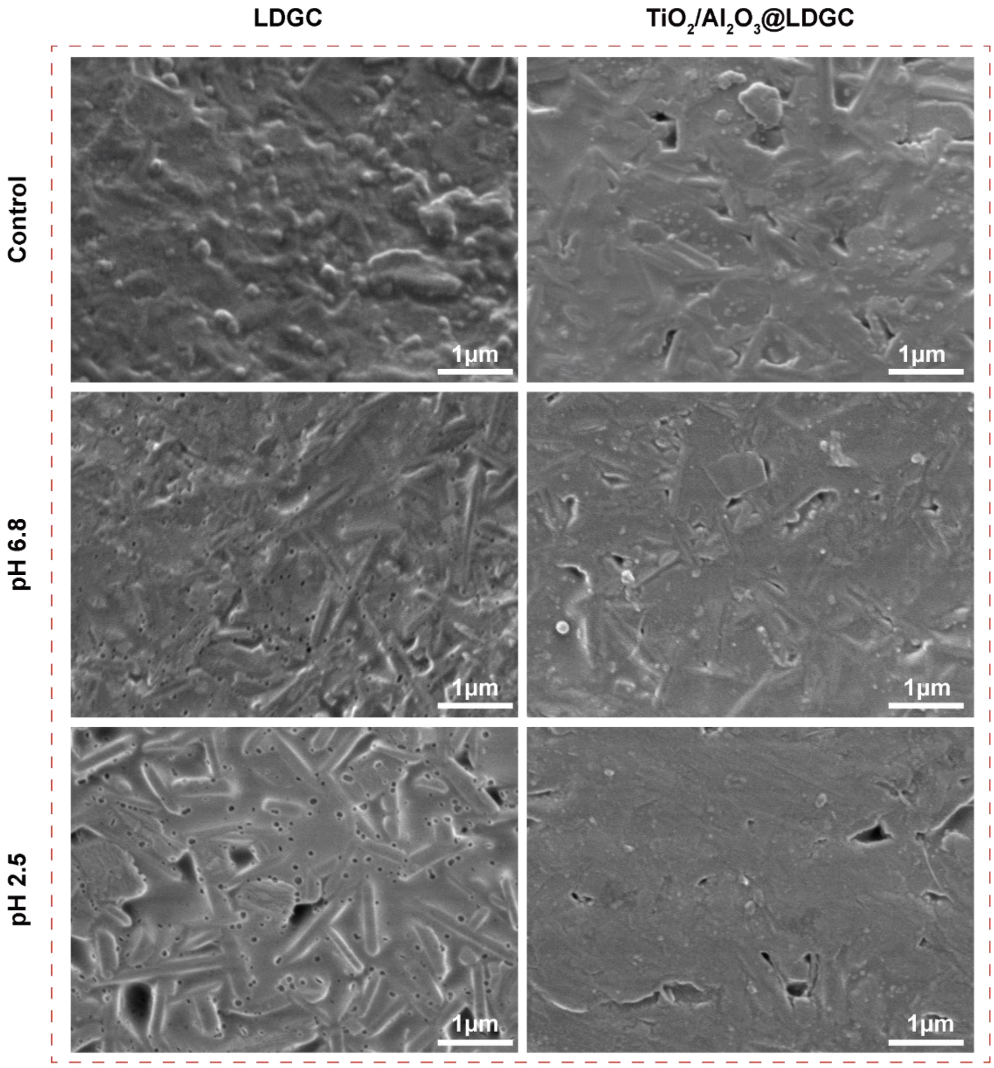

Supplementary Figure 9 | SEM images of the veneer with or without TiO_2/Al_2O_3 film (UP. CAD) before and after immersion in the liquid environment treatment under different pH values.

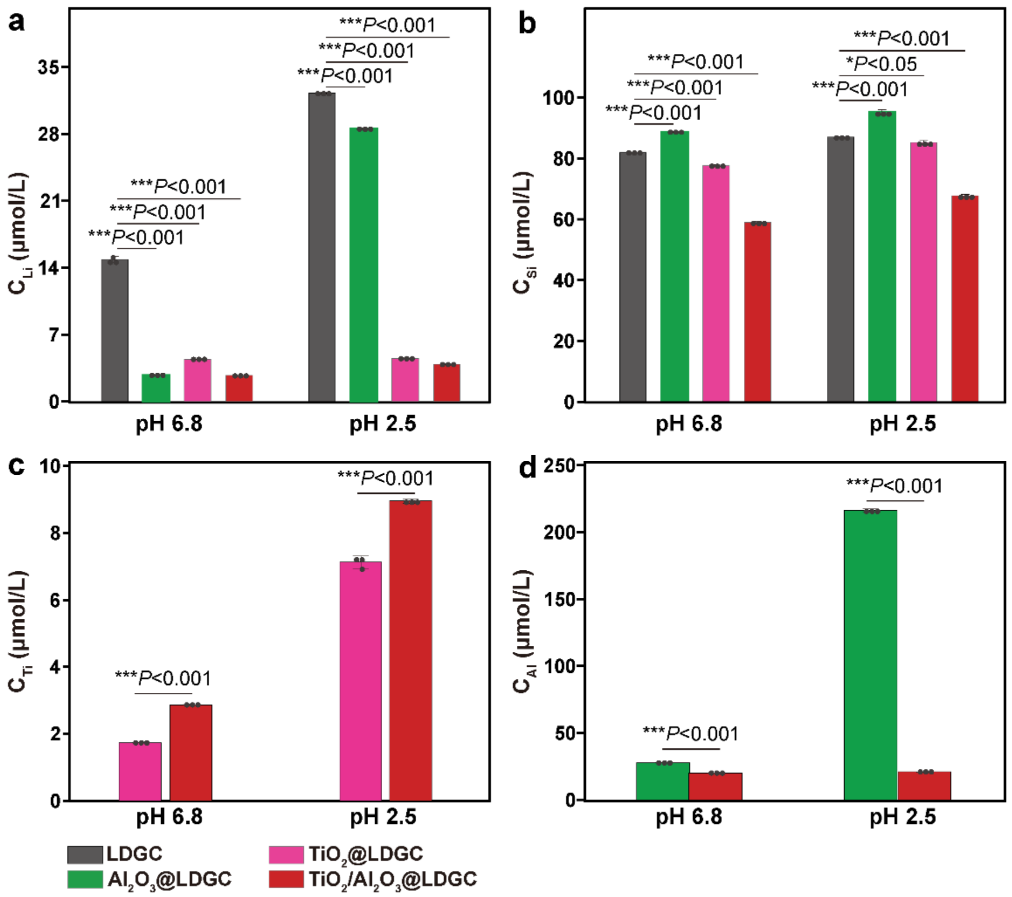

Supplementary Figure 10 | Concentration of the element released in solution (IPS e.max CAD). (a) Li element. Statistical significance was analyzed using one-way ANOVA with Tukey's multiple comparisons test. *P*(LDGC-Al_2O_3@LDGC) < 0.001, *P*(LDGC-TiO_2@LDGC) < 0.001, *P*(LDGC-TiO_2/Al_2O_3@LDGC) < 0.001; (b) Si element. Statistical significance was analyzed using one-way ANOVA with Tukey's multiple comparisons test. For pH 6.8, *P*(LDGC-Al_2O_3@LDGC) < 0.001, *P*(LDGC-TiO_2@LDGC) < 0.001, *P*(LDGC-TiO_2/Al_2O_3@LDGC) < 0.001; For pH 2.5, *P*(LDGC-Al_2O_3@LDGC) < 0.001, *P*(LDGC-TiO_2@LDGC) < 0.05, *P*(LDGC-TiO_2/Al_2O_3@LDGC) < 0.001; (c) Ti element. Statistical significance was analyzed using a t-test. *P*(TiO_2@LDGC-TiO_2/Al_2O_3@LDGC) < 0.001; (d) Al element. Statistical significance was analyzed using a t-test. *P*(Al_2O_3@LDGC-TiO_2/Al_2O_3@LDGC) < 0.001; Data are presented as mean ± s.d. of *n* = 3 biological replicates. Error bars represent s.d.. Source data are provided as a Source Data file.

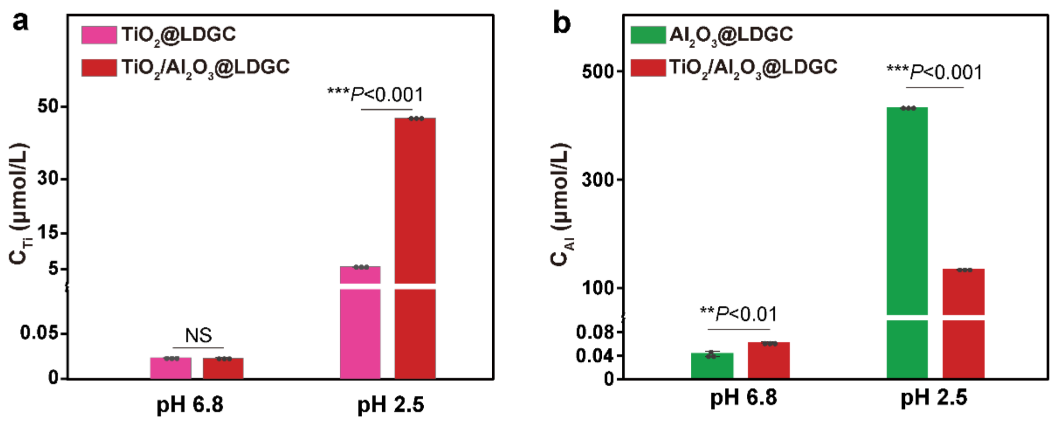

Supplementary Figure 11 | Concentration of the element released in solution (UP. CAD). (a) Ti element. Statistical significance was analyzed using a t-test. NS is not significant. For pH 2.5, $P(TiO_2@LDGC\text{-}TiO_2/Al_2O_3@LDGC) < 0.001$; (b) Al element. Statistical significance was analyzed using a t-test (n = 3). For pH 6.8, $P(Al_2O_3@LDGC\text{-}TiO_2/Al_2O_3@LDGC) < 0.01$; For pH 2.5, $P(Al_2O_3@LDGC\text{-}TiO_2/Al_2O_3@LDGC) < 0.001$; Data are presented as mean ± s.d. of $n = 3$ biological replicates. Error bars represent s.d.. Source data are provided as a Source Data file.

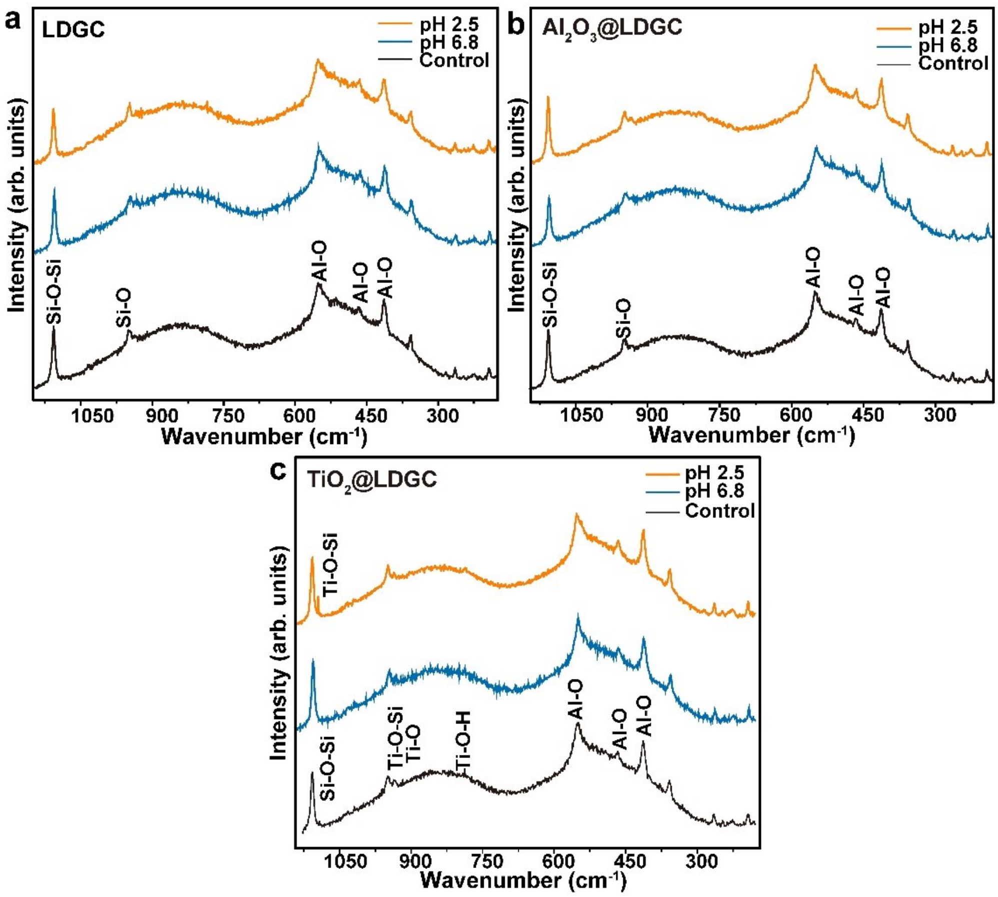

Supplementary Figure 12 | Raman spectra of the samples that were untreated (control), and immersed in artificial saliva (at pH 6.8 and 2.5), respectively. (a) LDGC; (b) Al_2O_3@LDGC; (c) and TiO_2@LDGC. Source data are provided as a Source Data file.

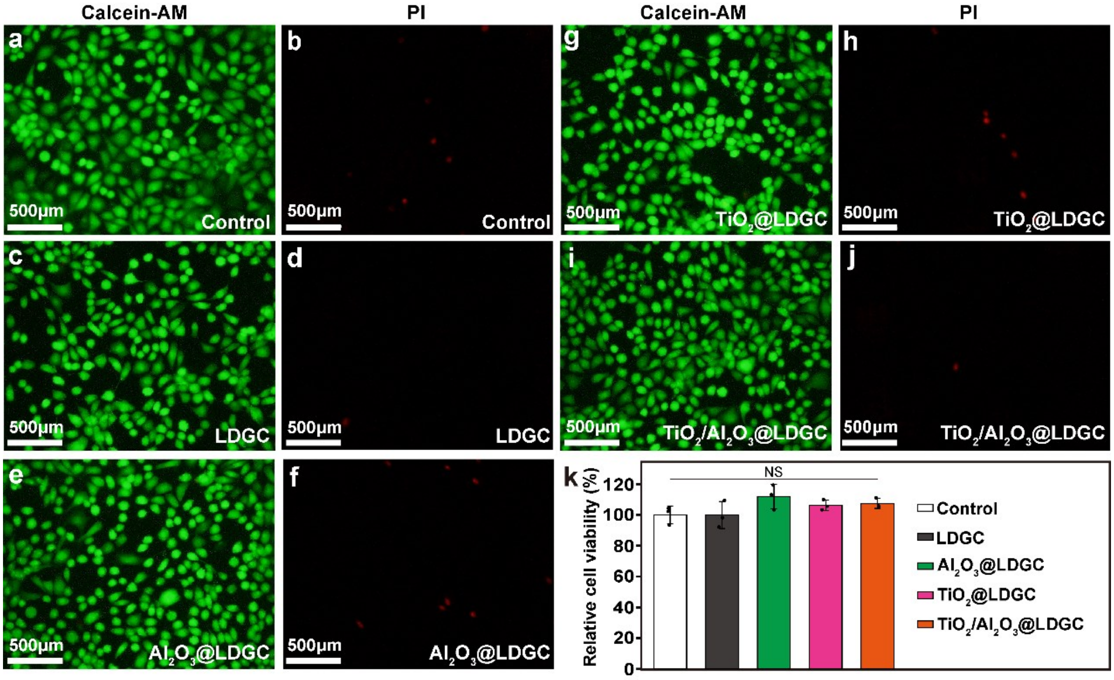

Supplementary Figure 13 | Cytotoxicity test of fibroblasts after 48 hours of treatment. Fluorescence images show the live cells (green) and dead cells (red) on the tested surfaces that were cultured in (a, b) culture medium (control); and extracts of (c, d) LDGC; (e, f) Al_2O_3@LDGC; (g, h) TiO_2@LDGC; (i, j) TiO_2/Al_2O_3@LDGC, respectively. (k) Cell viability assay of fibroblasts was assessed using the CCK-8 assay in culture medium and immersion solution of LDGC, Al_2O_3@LDGC, TiO_2@LDGC, TiO_2/Al_2O_3@LDGC, respectively. Statistical significance was analyzed using one-way ANOVA with Tukey's multiple comparisons test. NS is not significant. Data are presented as mean ± s.d. of $n = 3$ biological replicates. Error bars represent s.d.. Source data are provided as a Source Data file.

Supplementary Table 1. The Si/O atomic ratio in samples of LDGC cross-section detected by EDS.

Sample	Si (%)	O (%)	Si/O
TiO_2/Al_2O_3@LDGC	16.04	44.65	0.359
pH 2.5 TiO_2/Al_2O_3@LDGC	27.38	48.39	0.566
pH 2.5 LDGC	33.29	24.28	1.371

Supplementary Table 2. Compositions of the DMEM.

Components	Molecular Weight	Concentration (mg/L)	mM
**Amino Acids**			
Glycine	75.0	30.0	0.4
L-Arginine hydrochloride	211.0	84.0	0.39810428
L-Cystine 2HCl	313.0	63.0	0.20127796
L-Glutamine	146.0	584.0	4.0
L-Histidine hydrochloride-H_2O	210.0	42.0	0.2
L-Isoleucine	131.0	105.0	0.8015267
L-Leucine	131.0	105.0	0.8015267
L-Lysine hydrochloride	183.0	146.0	0.7978142
L-Methionine	149.0	30.0	0.20134228
L-Phenylalanine	165.0	66.0	0.4
L-Serine	105.0	42.0	0.4
L-Threonine	119.0	95.0	0.79831934
L-Tryptophan	204.0	16.0	0.078431375
L-Tyrosine disodium salt dihydrate	261.0	104.0	0.39846742
L-Valine	117.0	94.0	0.8034188
**Vitamins**			
Choline chloride	140.0	4.0	0.028571429
D-Calcium pantothenate	477.0	4.0	0.008385744
Folic Acid	441.0	4.0	0.009070295
Niacinamide	122.0	4.0	0.032786883
Pyridoxine hydrochloride	206.0	4.0	0.019417476
Riboflavin	376.0	0.4	0.0010638298

Components	Molecular Weight	Concentration (mg/L)	mM
Thiamine hydrochloride	337.0	4.0	0.011869436
i-Inositol	180.0	7.2	0.04
**Inorganic Salts**			
Calcium Chloride ($CaCl_2$) (anhyd.)	111.0	200.0	1.8018018
Ferric Nitrate ($Fe(NO_3)_3$"$9H_2O$)	404.0	0.1	2.4752476E-4
Magnesium Sulfate ($MgSO_4$) (anhyd.)	120.0	97.67	0.8139166
Potassium Chloride (KCl)	75.0	400.0	5.3333335
Sodium Bicarbonate ($NaHCO_3$)	84.0	3700.0	44.04762
Sodium Chloride (NaCl)	58.0	6400.0	110.344826
Sodium Phosphate monobasic (NaH_2PO_4-H_2O)	138.0	125.0	0.9057971
**Other Components**			
D-Glucose (Dextrose)	180.0	4500.0	25.0
Phenol Red	376.4	15.0	0.039851222
Sodium Pyruvate	110.0	110.0	1.0
