# Supplementary material for: Hybrid TiO2/Al2O3 nanolayer overcoating enhances dental lithium disilicate glass-ceramics acid resistance and surface mechanical properties
Source: Nat Commun. 2025 Nov 21;16:11530. doi: 10.1038/s41467-025-66560-8 (PMC12748710; doi:10.1038/s41467-025-66560-8)
Supplement: Supplementary file 2 — Reporting summary [file 41467_2025_66560_MOESM2_ESM.pdf]

nature portfolio

Corresponding author(s): Bin Zhang, Yong Qin, Xing Wang

Last updated by author(s): Sep 13, 2025

Reporting Summary

Nature Portfolio wishes to improve the reproducibility of the work that we publish. This form provides structure for consistency and transparency in reporting. For further information on Nature Portfolio policies, see our Editorial Policies and the Editorial Policy Checklist.

Statistics

For all statistical analyses, confirm that the following items are present in the figure legend, table legend, main text, or Methods section.

n/a	Confirmed	
☐	☒	The exact sample size (*n*) for each experimental group/condition, given as a discrete number and unit of measurement
☐	☒	A statement on whether measurements were taken from distinct samples or whether the same sample was measured repeatedly
☐	☒	The statistical test(s) used AND whether they are one- or two-sided<br>*Only common tests should be described solely by name; describe more complex techniques in the Methods section.*
☒	☐	A description of all covariates tested
☒	☐	A description of any assumptions or corrections, such as tests of normality and adjustment for multiple comparisons
☒	☐	A full description of the statistical parameters including central tendency (e.g. means) or other basic estimates (e.g. regression coefficient) AND variation (e.g. standard deviation) or associated estimates of uncertainty (e.g. confidence intervals)
☒	☐	For null hypothesis testing, the test statistic (e.g. *F*, *t*, *r*) with confidence intervals, effect sizes, degrees of freedom and *P* value noted<br>*Give P values as exact values whenever suitable.*
☒	☐	For Bayesian analysis, information on the choice of priors and Markov chain Monte Carlo settings
☒	☐	For hierarchical and complex designs, identification of the appropriate level for tests and full reporting of outcomes
☒	☐	Estimates of effect sizes (e.g. Cohen's *d*, Pearson's *r*), indicating how they were calculated

Our web collection on statistics for biologists contains articles on many of the points above.

Software and code

Policy information about availability of computer code

Data collection	no software was used
Data analysis	Oringin 2025, GraphPad Prism version 8.0

For manuscripts utilizing custom algorithms or software that are central to the research but not yet described in published literature, software must be made available to editors and reviewers. We strongly encourage code deposition in a community repository (e.g. GitHub). See the Nature Portfolio guidelines for submitting code & software for further information.

Data

Policy information about availability of data

All manuscripts must include a data availability statement. This statement should provide the following information, where applicable:

- Accession codes, unique identifiers, or web links for publicly available datasets
- A description of any restrictions on data availability
- For clinical datasets or third party data, please ensure that the statement adheres to our policy

Provide your data availability statement here.

Research involving human participants, their data, or biological material

Policy information about studies with human participants or human data. See also policy information about sex, gender (identity/presentation), and sexual orientation and race, ethnicity and racism.

Reporting on sex and gender	This information has not been collected.
Reporting on race, ethnicity, or other socially relevant groupings	This information has not been collected.
Population characteristics	See above
Recruitment	This study has strict inclusion and exclusion criteria for participants, and the following inclusion criteria were used: (1) patients need to undergo aesthetic restoration of two or more front teeth at the same time; (2) patients voluntarily participate in clinical trials with good compliance and regular follow-up; (3) patients aged between 20-40 with no missing teeth. The exclusion criteria were: (1) patients with jaw fractures, chronic systemic diseases (diabetes, cardiovascular diseases, etc.), smoking habits; (2) Pregnant women; (3) Patients with chronic periodontal disease.It is not necessary to include vulnerable subjects (such as minors, pregnant women, prisoners, students, illiterates, persons with limited or no capacity). If patients experience physical discomfort or do not wish to continue participating in the study, they may withdraw from the study.<br>Collecting photos only shows the appearance of the veneer and does not involve any data bias.
Ethics oversight	Medical Ethics Committee of Shanxi Medical University Stomatological Hospital (Approval No. 2024SLL002)

Note that full information on the approval of the study protocol must also be provided in the manuscript.

Field-specific reporting

Please select the one below that is the best fit for your research. If you are not sure, read the appropriate sections before making your selection.

☒ Life sciences ☐ Behavioural & social sciences ☐ Ecological, evolutionary & environmental sciences

For a reference copy of the document with all sections, see nature.com/documents/nr-reporting-summary-flat.pdf

Life sciences study design

All studies must disclose on these points even when the disclosure is negative.

Sample size	No statistical method was used to determine sample size; sample sizes were chosen based on the standard in the field.
Data exclusions	No data was excluded.
Replication	Each experiment was reproduced at least three times on separate occasions. Experimental findings were reliably reproduced.
Randomization	Samples were not randomized because these studies involved defined molecular reagents used in specific mechanistic assays. Randomization is not standard in this field. This study did not involve animals or human research participants.
Blinding	Blinding was not necessary because experimental conditions were well-controlled and experimental results were quantitative and did not require subjective interpretation or analysis. Blinding is not standard in this field. This study did not involve animals or human research participants

Reporting for specific materials, systems and methods

We require information from authors about some types of materials, experimental systems and methods used in many studies. Here, indicate whether each material, system or method listed is relevant to your study. If you are not sure if a list item applies to your research, read the appropriate section before selecting a response.

Materials & experimental systems

n/a	Involved in the study
☒	☐ Antibodies
☐	☒ Eukaryotic cell lines
☒	☐ Palaeontology and archaeology
☒	☐ Animals and other organisms
☒	☐ Clinical data
☒	☐ Dual use research of concern
☒	☐ Plants

Methods

n/a	Involved in the study
☒	☐ ChIP-seq
☒	☐ Flow cytometry
☒	☐ MRI-based neuroimaging

Eukaryotic cell lines

Policy information about cell lines and Sex and Gender in Research

Cell line source(s): L929

Authentication: The authentication of L929 cells was performed as follows: Genomic DNA was extracted using the TIANamp Genomic DNA Kit. A total of 20 STR loci and sex-determining loci were amplified using the Microreader™21 ID System. PCR products were detected by the GenReader7010 Genetic Analyzer (Zhiyue Gene), and the results were analyzed with GeneMapper Software 6 (Applied Biosystems). The STR profiles were then compared against the ExPASy database to confirm cell identity.

Mycoplasma contamination: the cell lines were not tested for mycoplasma contamination

Commonly misidentified lines (See ICLAC register): No commonly misidentified cell lines were used in the study.

Plants

Seed stocks: No plants were used in this study

Novel plant genotypes: No plants were used in this study

Authentication: No plants were used in this study
